# Clinicopathological features of adult lymphoblastic lymphoma: a retrospective multicenter study from Türkiye

**DOI:** 10.3389/fonc.2026.1820823

**Published:** 2026-05-18

**Authors:** Cem Selim, Rafiye Çiftçiler, Deniz Gören, Kemal Fidan, Ali Ünal, Hacer Berna Afacan Öztürk, Murat Albayrak, Meral Uluköylü Mengüç, Işık Atagündüz, Emel İşleyen, Gülsüm Özet, Elif Birtaş Ateşoğlu

**Affiliations:** 1 Adult Hematology Department, Practice and Research Hospital, Konya Selçuk University Faculty of Medicine, Konya, Türkiye; 2 Adult Hematology Department, Şişli Memorial Hospital, Istanbul, Türkiye; 3 Adult Hematology Department, Kayseri City Hospital, Kayseri, Türkiye; 4 Adult Hematology Department, Erciyes University Faculty of Medicine Hospital, Kayseri, Türkiye; 5 Adult Hematology Department, Etlik City Hospital, Ankara, Türkiye; 6 Adult Hematology Department, Marmara University Faculty of Medicine Hospital, Istanbul, Türkiye; 7 Adult Hematology Department, Bilkent City Hospital, Ankara, Türkiye; 8 Adult Hematology Department, Practice and Research Hospital, Yeditepe University Faculty of Medicine, Istanbul, Türkiye

**Keywords:** diagnosis, follow-up, *Lymphoblastic lymphoma*, outcomes, risk factors, survival, treatment

## Abstract

**Introduction:**

Lymphoblastic lymphoma (LBL) is a highly aggressive malignancy composed of immature lymphocytes of B- cell or T-cell origin. LBL is typically distinguished from acute lymphoblastic leukemia (ALL) by the presence of fewer than 20% bone marrow blasts. This retrospective multicenter cohort study evaluated the clinical data of adult LBL patients diagnosed and treated at tertiary centers in Türkiye.

**Materials and methods:**

Thirty-eight patients from seven tertiary adult hematology clinics were included. Eligible patients were ≥18 years of age, had a pathologically confirmed diagnosis of LBL, underwent radiological staging, and received at least one chemotherapy regimen.

**Results:**

The median overall survival (OS) for the entire cohort was 23 months. No statistically significant difference in OS was observed between patients with T-LBL and those with B-LBL (p>0.05). The median progression-free survival (PFS) was 6 months. The 5-year OS rate was 6%, underscoring the aggressive nature of adult LBL.

**Discussion:**

Our findings **suggest exploratory univariate associations** between certain clinical features and inferior OS. Elevated LDH levels, central nervous system involvement, disease affecting ≥3 anatomical regions, cervical lymphadenopathy, failure to achieve remission after initial therapy, and male gender may correlate with poorer outcomes. These results should be interpreted cautiously given the limited sample size and reliance on univariate analyses.

## Introduction

Lymphoblastic lymphoma (LBL) is a highly malignant neoplasm composed of immature lymphocytes derived from B or T cells and, less frequently, from natural killer (NK) cells. It shares biological and morphological features with acute lymphoblastic leukemia (ALL). Although the 2017 and 2022 WHO classifications group these entities together, LBL is typically distinguished from ALL by the presence of fewer than 20% marrow-infiltrating blasts (1, 2). Extra-organ involvement and lymphadenopathy are more common in LBL than in ALL and carry prognostic significance (2). Despite their biological similarities, LBL and ALL should be regarded as clinically distinct entities, given the substantial differences in clinical presentation, prognostic variables, and therapeutic strategies (3).

LBL is a rare disease for which precise incidence data remain limited. In earlier epidemiological studies, LBL was frequently categorized together with ALL (estimated incidence 1.3 per 100,000 annually) or Burkitt lymphoma (1.46 per 100,000 annually) (4). In adults, LBL accounts for approximately 2–4% of non-Hodgkin lymphomas or ALL cases, whereas in children it represents less than 30% of cases. The apparent prevalence of LBL has increased in parallel with heightened disease awareness. LBL is most commonly diagnosed in young males aged 10–30 years and, unlike ALL, more frequently exhibits a T-cell immunophenotype, leading to greater lymph node and solid organ involvement (4, 5).

B-lymphoblastic lymphoma (B-LBL) is consistently positive for at least two B-cell markers, typically CD19, CD79, and CD22. CD10, CD24, PAX5, and TdT are expressed in the majority of cases, whereas CD20 and the stem cell antigen CD34 show variable expression, and CD45 may be absent (6). T-lymphoblastic lymphoma (T-LBL) frequently exhibits a cortical CD1a+ phenotype; early-T phenotypes are uncommon, associated with a lower risk of mediastinal involvement but a higher risk of bone marrow dissemination. An early thymic precursor (ETP) phenotype, initially described in T-ALL, is occasionally observed in LBL. These cases express very early T-lymphoid markers without CD1a, CD8, and CD antigens (CD5-negative or dim) and typically co-express stem/myeloid cell markers (7).

The International Prognostic Index (IPI), incorporating age, extranodal involvement, LDH levels, stage, and performance status, has not proven predictive for LBL. Historically, patients with stage IV disease, bone marrow or CNS involvement, and elevated LDH levels (>300 IU/L) demonstrated only a 19% five-year relapse-free survival, compared with 94% in low-risk patients (8). More recent studies indicate that older age, Black ethnicity, and advanced stage may further worsen prognosis.

Genetic alterations such as t (9, 17)(q34;q32) translocation and loss of heterozygosity at 6q have been associated with adverse outcomes (9). The GRAAL-LYSA study proposed an oncogenetic-based prognostic score for T-LBL, in which *NOTCH1/FBXW7* mutations, in the absence of *RAS/PTEN* abnormalities, were linked to a favorable prognosis (10). Conversely, adult patients with ETP-LBL receiving standard chemotherapy exhibited significantly poorer outcomes compared with non-ETP cases (11). Moreover, CT/PET response assessment and evaluation of minimally disseminated disease (MDD) or measurable residual disease (MRD) may play a critical role in prognostication (3).

The implementation of intensive ALL-like protocols has led to substantial progress in the management of adult LBL (12). In addition, notable improvements have been achieved through diverse treatment strategies, including pediatric-inspired regimens, radiotherapy, allogeneic hematopoietic stem cell transplantation, and intrathecal therapy, particularly in patients at high risk of central nervous system involvement (13, 14).

Despite recent advances, consensus on prognostic factors and optimal treatment strategies for LBL remains limited, largely due to the rarity of the disease and the small patient population. To address this gap, we analyzed data from LBL patients diagnosed and treated across multiple centers in our country. Our objective was to identify potential risk factors and evaluate their impact on overall survival and progression-free survival.

## Materials and methods

### Patient selection

This retrospective study included 38 patients treated between 2018 and June 2024 across seven tertiary adult hematology clinics. Eligible patients were aged 18 years or older, had a confirmed diagnosis of lymphoblastic lymphoma (LBL) from lymph nodes or other extranodal sites, demonstrated fewer than 20% bone marrow blasts on biopsy, underwent imaging studies for staging, and received at least one cycle of chemotherapy. The primary endpoint of the study was overall mortality. Owing to its retrospective design, heterogeneity in treatment and follow-up protocols across participating centers contributed to data variability. Patients with incomplete records were excluded from subgroup analyses but retained in OS and PFS calculations; details are provided in **Supplementary Table 1**.

### Diagnostic standardization

All participating centers established the diagnosis of LBL through tissue biopsy and flow cytometry. A standardized immunophenotypic panel was applied, including CD3, CD20, TdT, CD10, and CD34, with additional markers (CD19, CD79a, PAX5 for B-LBL; CD1a, CD5, CD7 for T-LBL) used when indicated. All centers adhered to the WHO 2022 and ICC classification criteria. Bone marrow involvement was defined as <20% blasts at diagnosis, consistent with WHO criteria (1). Cases with subsequent marrow progression were documented accordingly. Although centralized pathology review was not performed, all tertiary centers followed national hematopathology guidelines to ensure methodological consistency.

### Evaluation and follow-up

All patients at the study centers underwent enhanced computed tomography (CT) or positron emission tomography/computed tomography (PET/CT) for evaluation and staging. Bone marrow biopsy was performed in all participants. Treatment response was assessed according to the updated efficacy criteria established by the International Working Group (IWG) for malignant lymphoma (15). Following therapy, patients in remission were monitored every three months during the first two years, every six months from years three to five, and annually thereafter. Patients not achieving remission were followed more frequently based on clinical and laboratory findings, with hospitalization arranged when necessary. At each follow-up visit, patients in remission were evaluated with complete blood counts, serum lactate dehydrogenase (LDH) levels, and imaging modalities such as ultrasonography when clinically indicated.

### Response assessment and statistical analysis

Treatment response was assessed according to the Cheson 1999 criteria, which remain widely applied in lymphoid malignancies and provide standardized definitions for remission and relapse. The use of this framework was justified by its established role in clinical trials and retrospective analyses of lymphoblastic lymphoma. For patients staged and monitored with PET/CT, metabolic response was evaluated using Deauville criteria, with visual assessment performed by experienced nuclear medicine specialists. PET/CT was not used uniformly across centers, and therefore a subset of patients were assessed by Cheson 1999 criteria alone. We acknowledge that this heterogeneity in response definitions represents a limitation of the study and may have introduced confounding in PFS calculations.

Procedures for handling missing data were predefined: patients with incomplete clinical or laboratory records were excluded from specific subgroup analyses but retained in overall survival calculations. Censoring rules were applied at the date of last follow-up for patients alive without progression. Overall survival (OS) was calculated from the date of diagnosis until death from any cause or last follow-up, while progression-free survival (PFS) was calculated from the date of diagnosis until disease progression, relapse, or death, whichever occurred first.

Statistical analyses were conducted using SPSS version 25 (IBM Corp., Armonk, NY, USA). Survival was assessed through univariate analyses employing the log-rank test, and survival rates were estimated using the Kaplan–Meier method. Given the limited sample size, only univariate analyses were performed; no Cox proportional hazards models were fitted. A Type I error threshold of <5% was considered statistically significant.

## Results

**Figure 1:**
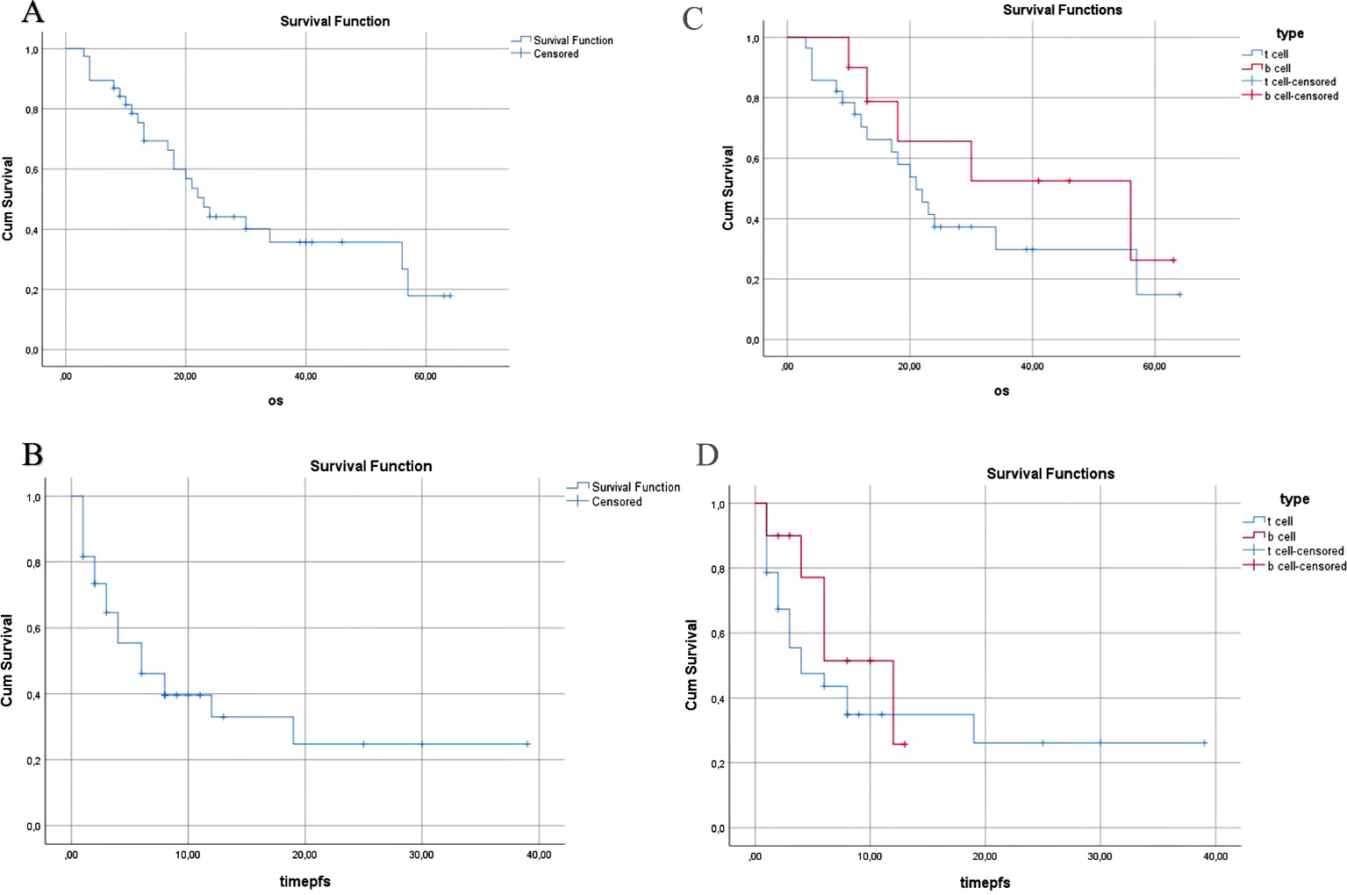
Kaplan–Meier curves for overall survival (OS) and progression-free survival (PFS) in adult LBL patients. **(A)** OS of the entire study population. **(B)** PFS of the entire study population. **(C)** OS by subtype (T-LBL vs B-LBL). **(D)** PFS by subtype (T-LBL vs B-LBL). Censored cases are indicated by tick marks. LBL, lymphoblastic lymphoma; T-LBL, T-cell lymphoblastic lymphoma; B-LBL, B-cell lymphoblastic lymphoma.

The median age of the 38 patients with LBL included in the study was 37 ± 13 years; 24 (63%) were male and 14 (37%) were female. The ages of patients diagnosed with T-LBL and B-LBL were statistically similar (p>0.05), and no significant association was observed between increasing age and overall survival (OS) (p>0.05). The median follow-up period was 23 months (range, 1–64 months). Among the 38 patients, 10 (26%) were diagnosed with B-LBL using tissue biopsy and flow cytometry, whereas 28 (74%) were classified as T-LBL based on the same diagnostic methods. The median OS was 23 months for all patients, 21 months for those with T-LBL, and 56 months for those with B-LBL. Despite the apparent disparity in median OS between patients with T-LBL and B-LBL, no statistically significant difference was detected, likely due to the limited number of patients with B-LBL (p>0.05). The median progression-free survival (PFS) was 6 months overall, 4 months in patients with T-LBL, and 12 months in those with B-LBL. No statistically significant difference was observed in PFS between the two groups. The 5-year OS rate for the entire cohort was 6%, reflecting the steep decline in survival after two years. **Figure 1** illustrates the Kaplan–Meier curves for OS and PFS.

Among patients diagnosed with T-LBL, 20 were male and 8 were female, whereas in the B-LBL group, 4 were male and 6 were female. Although women were more prevalent in the B-LBL cohort, no statistically significant difference in gender distribution was observed between the two groups (p>0.05). The median OS for men with T-LBL was 20 months compared to 24 months for women, with no significant difference between the groups (p>0.05). In contrast, the median OS for men with B-LBL was 13 months, whereas for women it was 56 months, revealing a statistically significant difference (p<0.05). When all patients with LBL were analyzed collectively, the median OS was 18 months for men and 56 months for women, again demonstrating a statistically significant difference (p<0.05). Laboratory values of the patients are presented in **Table 1**. Although median lymphocyte counts were higher in B-LBL compared to T-LBL (**Table 1**), this difference was not clinically meaningful and did not correlate with survival outcomes.

**Table 1:** Baseline clinical and laboratory characteristics of patients with LBL, T-LBL, and B-LBL.

Parameter	LBL (n=38)	T-LBL (n=28)	B-LBL (n=10)
Age (years), median (IQR)	37 (30–45)	38 (31–46)	34 (28–40)
Sedimentation (mm/h), median (IQR)	43 (25–60)	49 (30–65)	26 (20–35)
LDH (U/L), median (range)	604 (300–1200)	660 (320–1300)	440 (280–800)
Platelet (×10³/µL), mean ± SD	213,800 ± 45,000	200,000 ± 40,000	250,800 ± 50,000
WBC (cells/µL), median (IQR)	8,970 (5,000–15,000)	8,990 (5,200–14,800)	5,050 (3,000–8,000)
Lymphocyte count (cells/µL), median (IQR)	3,320 (2,000–5,000)	3,800 (2,200–5,500)	4,300 (2,500–6,000)
Hemoglobin (g/dl), mean ± SD	12.3 ± 1.2	12.0 ± 1.1	13.3 ± 1.3

LBL, lymphoblastic lymphoma; T-LBL, T-cell lymphoblastic lymphoma; B-LBL, B-cell lymphoblastic lymphoma; OS, overall survival; LDH, lactate dehydrogenase; WBC, white blood cell count.

Regarding disease characteristics, 19 patients (50%) were diagnosed via mediastinal lymph node biopsy and 10 (26%) via cervical lymph node biopsy. Mediastinal lymph node involvement was present in 34 patients (89.5%), while 30 patients (79%) had cervical lymph node involvement. Pleural or pericardial effusion was detected in 29 patients (76%), and bone marrow involvement in 21 patients (55%). Central nervous system involvement was observed in 6 patients (16%), involvement of three or more anatomical regions in 22 patients (58%), and extra-organ involvement in 19 patients (50%).

Twenty-nine patients (76% of the cohort) were classified as stage IV. Four patients were classified as Stage I–II, which is atypical for LBL. These rare cases may reflect early detection in localized nodal presentations, consistent with prior reports. Ten patients (26%) underwent autologous stem cell transplantation (auto-SCT), 11 (29%) underwent allogeneic stem cell transplantation (allo-SCT), and 17 (45%) did not receive transplantation. Second-line therapy was administered to 28 patients (73.7%), and 18 patients (47%) required third-line treatment. The median overall survival (OS) of the cohort was 23 months. Baseline clinical characteristics of the patients are summarized in **Table 2**.

**Table 2:** Clinical characteristics, treatment, and outcomes of patients with LBL, T-LBL, and B-LBL.

Parameter		Category	LBL (n=38)	T-LBL (n=28)	B-LBL (n=10)
Gender n (%)		Male	24 (63%)	20 (71%)	4 (40%)
		Female	14 (37%)	8 (29%)	6 (60%)
Lymphadenopathy (LAP) n (%)		Cervical	30 (79%)	25 (89%)	5 (50%)
		Mediastinal	34 (89.5%)	25 (89%)	9 (90%)
		Axillary	15 (39.5%)	13 (46%)	2 (20%)
		Supraclavicular	12 (32%)	10 (36%)	2 (20%)
		Abdominal	16 (42%)	14 (50%)	2 (20%)
		Inguinal LAP	9 (24%)	8 (29%)	1 (10%)
Pleural/Pericardial fluid n (%)			29 (76%)	22 (79%)	7 (70%)
Bone marrow involvement n (%)			21 (55%)	19 (68%)	2 (20%)
CNS involvement n (%)			6 (16%)	5 (18%)	1 (10%)
≥3 anatomical regions involved n (%)			22 (58%)	17 (61%)	5 (50%)
Extra-organ involvement n (%)			19 (50%)	11 (39%)	8 (80%)
Immunophenotype n (%)		T cell marker	28 (74%)	28 (100%)	–
		B cell marker	10 (26%)	–	10 (100%)
		Myeloid marker	6 (16%)	3 (11%)	3 (30%)
Stage n (%)		I	4 (11%)	2 (7%)	2 (20%)
		II	2 (5%)	2 (7%)	0
		III	3 (8%)	2 (7%)	1 (10%)
		IV	29 (76%)	22 (79%)	7 (70%)
First line treatment n (%)	EPOCH		12 (31.6%)	10 (36%)	2 (20%)
	CHOP		5 (13%)	3 (11%)	2 (20%)
	HYPERCVAD		11 (29%)	7 (25%)	4 (40%)
	BFM		3 (8%)	1 (4%)	2 (20%)
	GMALL		3 (8%)	3 (11%)	0
	CALGB		2 (5%)	2 (7%)	0
	CHOEP		2 (5%)	2 (7%)	0
Response to first-line therapy n (%)	Complete Remission		13 (34%)	9 (32%)	4 (40%)
	Partial remission		15 (40%)	12 (43%)	3 (30%)
	Stable disease		3 (8%)	3 (11%)	0
	Progressive disease		7 (18%)	4 (14%)	3 (30%)
Transplantation n (%)	Auto-SCT		10 (26%)	7 (25%)	3 (30%)
	Allo-SCT		11 (29%)	9 (32%)	2 (20%)
	None		17 (45%)	12 (43%)	5 (50%)
Patient status n (%)	Alive at follow-up		15 (40%)	10 (36%)	5 (50%)
	Deceased		23 (60.5%)	18 (64%)	5 (50%)

Auto-SCT, autologous stem cell transplantation; Allo-SCT, allogeneic stem cell transplantation; LAP, lymphadenopathy; CNS, central nervous system; LBL, lymphoblastic lymphoma; T-LBL, T-cell lymphoblastic lymphoma; B-LBL, B-cell lymphoblastic lymphoma.

Bone marrow involvement refers to infiltration by lymphoblasts below the 20% threshold, consistent with WHO 2022 and ICC diagnostic criteria for lymphoblastic lymphoma (LBL). Patients with ≥20% blasts were excluded as acute lymphoblastic leukemia (ALL).

Twelve (31.6%) of the patients in the study received the EPOCH (etoposide, doxorubicin, cyclophosphamide, vincristine, and prednisone) protocol at their own centers, and 9 of the 12 patients completed the treatment. Three of the patients who received EPOCH treatment received autologous hematopoietic stem cell transplantation (auto-SCT), and the other received allogeneic hematopoietic stem cell transplantation (allo-SCT). Five (14%) of the patients in the centers participating in the study received CHOP (doxorubicin, cyclophosphamide, vincristine, and prednisone) treatment. Four of the patients who received CHOP treatment were able to complete the treatment, and one could not complete the treatment due to disease progression. Two of the patients who received CHOP treatment received allogeneic stem cell transplantation. CHOP was selected in these cases because the patients were considered unfit for intensive regimens due to comorbidities or poor performance status, and therefore received a less intensive protocol. Eleven (30%) patients received the HYPERCVAD (doxorubicin, cyclophosphamide, vincristine, dexamethasone, methotrexate, and cytarabine) protocol, and all patients who received this treatment completed the treatment. Four of these patients had auto-SCT, and four had allo-SCT. Three (7%) of the patients included in the study received the BFM (doxorubicin, cyclophosphamide, vincristine, dexamethasone, methotrexate, cytarabine, L-asparaginase, 6-mercaptopurine) protocol; two of these patients completed the treatment, and the patients who completed the treatment underwent auto-SCT. Three patients (7%) received the GMALL (daunorubicin, cyclophosphamide, vincristine, dexamethasone, methotrexate, cytarabine, L-asparaginase, and 6-mercaptopurine) protocol, and two patients who completed treatment underwent allo-SCT, and one patient underwent auto-SCT. Two patients (5%) received CALGB (daunorubicin, cyclophosphamide, vincristine, prednisolone, methotrexate, cytarabine, pegasparaginase, and 6-mercaptopurine) treatment, and one patient who completed treatment underwent allo-SCT, and one patient was followed up without transplantation. Finally, two patients (5%) received CHOEP (etoposide, doxorubicin, cyclophosphamide, vincristine, and prednisone) treatment, and two patients who completed treatment were followed up without transplantation.

The treatments received by the 38 patients included in the study were summarized. Median OS values according to treatment regimens are presented in **Table 3**. These data are descriptive in nature and should not be interpreted as direct comparisons of efficacy, given the heterogeneity of treatment modalities, center-specific practices, and patient characteristics. No statistically significant differences were observed among regimens.

**Table 3:** Median overall survival (OS) according to treatment received (values are descriptive; direct comparisons across regimens are not appropriate due to heterogeneity in patient characteristics and treatment practices; these figures should not be interpreted as comparative outcomes and are provided only for descriptive purposes.

	LBL median OS (month)	T-LBL median OS (month)	B-LBL median OS (month)
EPOCH	23	22	30
CHOP	20	18	56
HYPERCVAD	57	24	NR
BFM	10	9	10
GMALL	21	21	NR
CALGB	34	34	NR
CHOEP	17	17	NR
Total	23	21	56

LBL, lymphoblastic lymphoma; T-LBL, T-cell lymphoblastic lymphoma; B-LBL, B-cell lymphoblastic lymphoma. Overall survival (OS) could not be calculated (NR) for three patients in the B-LBL cohort treated with the HYPERCVAD regimen, as they remained alive at the time of analysis.

Among the patients in the study, 10 underwent autologous stem cell transplantation (7 with T-LBL and 3 with B-LBL), whereas 11 received allogeneic stem cell transplantation (9 with T-LBL and 2 with B-LBL), resulting in a total of 21 individuals who underwent transplantation. When assessing auto-SCT and allo-SCT patients independently, median OS was determined to be 23 months for auto-SCT patients and 53 months for allo-SCT patients. Despite the median OS of patients who underwent allogeneic stem cell transplantation (allo-SCT) being 53 months, the median OS of transplanted patients was not statistically substantially greater (p>0.05). Nevertheless, when auto-SCT and allo-SCT patients were assessed individually, no significant difference in median OS was seen (p>0.05).

Thirteen patients (34%) achieved remission after the first course of treatment, 15 patients (39%) had partial remission, 3 patients (8%) had stable disease, and 7 patients (19%) had progressive disease. Nine of the 13 patients who achieved remission were diagnosed with T-LBL and 4 with B-LBL. The proportion of patients with T-LBL and B-LBL who entered remission was statistically similar (p>0.05). The median OS was 57 months in patients who achieved complete remission after first-line treatment, 21 months in patients in partial remission, 13 months in those with stable disease, and 10 months in those with progressive disease. Median OS was significantly higher in patients with both T-LBL and B-LBL who entered remission after the first course of treatment compared to those who did not (p<0.05). Twenty-eight patients (74%) required second-line therapy because of disease progression. Three patients (8%) were refractory to first-line therapy and died. Eighteen patients (47%) received at least third-line therapy.

Among the patients in the study, 22 (58%) exhibited involvement of three or more lymph nodes or organs, whereas 16 (42%) did not show such involvement. The median overall survival (OS) was 18 months for patients with involvement of ≥3 anatomical regions, compared to 56 months for those without. Statistically, the presence of involvement in three or more distinct anatomical regions was associated with significantly reduced survival (p<0.05). Seventeen patients with ≥3 sites of involvement were classified in the T-LBL subgroup, whereas five were categorized in the B-LBL subgroup. Although the frequency of ≥3 site involvement was similar between the two subgroups (p>0.05), median OS appeared lower in both groups when ≥3 sites were affected, suggesting a possible association with inferior survival (p=0.032). Given the small subgroup sizes, this finding should be interpreted with caution.

Bone marrow involvement was identified in 21 patients (55%), whereas it was absent in 17 (45%). The median overall survival (OS) of patients with bone marrow involvement was 22 months, compared to 30 months in those without, with no statistically significant difference observed (p>0.05). Bone marrow involvement was present in 19 patients with T-LBL and in 2 patients with B-LBL, with no significant difference in frequency or OS between the two groups (p>0.05).

Nineteen patients (50%) exhibited extra-organ involvement beyond lymph nodes and bone marrow, with a median OS of 18 months compared to 33 months in those without. Despite this variation, no statistically significant difference was observed (p>0.05). Eleven patients with extra-organ involvement were diagnosed with T-LBL, whereas eight were diagnosed with B-LBL. No significant difference was observed in the prevalence of extra-organ involvement between the two subtypes (p>0.05), nor in OS among patients with extra-organ involvement in either group (p>0.05).

Median OS was 11 months in six patients (16%) with CNS involvement compared to 30 months in those without, suggesting a possible association with inferior survival (p=0.047). Given the limited number of patients, this observation should be regarded as exploratory. Five patients with CNS involvement were diagnosed with T-LBL and one with B-LBL. Statistical analysis indicated that CNS involvement was more frequent in the T-LBL subgroup (p<0.05), and OS appeared lower in this group, though the small sample size limits firm conclusions.

Cervical lymphadenopathy was identified in 30 patients (79%), with a median OS of 23 months. Among these, 25 patients were diagnosed with T-LBL (median OS 21 months) and 5 with B-LBL (median OS 56 months). While a difference in median OS was observed between the two groups (p=0.041), this finding should be interpreted cautiously given the small number of B-LBL patients. Overall, the presence of cervical lymphadenopathy was linked to shorter OS compared to patients without cervical involvement, whereas lymphadenopathy at other sites did not appear to impact survival. The distribution of lymphadenopathy was statistically comparable between the T-LBL and B-LBL subtypes. Factors influencing median OS are summarized in **Table 4**.

**Table 4:** Median overall survival (OS) according to clinical parameters in adult LBL patients.

Clinical parameter	Group	n	Events (deaths)	Median OS (months) [95% CI]	T-LBL median OS (month)	B-LBL median OS (month)	p-value
Extra-organ involvement	Yes	19	14	18 [12–24]	17	20	p=0.27
	No	19	9	33 [25–41]	30	33	Reference
Transplantation	Auto-SCT	10	7	23 [15–31]	21	30	p=0.41
	Allo-SCT	11	8	53 [38–68]	24	56	p=0.19
	None	17	8	17 [12–22]	17	32	Reference
≥3 anatomical regions	Yes	22	17	18 [13–23]	18	18	p=0.032
	No	16	6	56 [42–70]	56	56	Reference
Bone marrow involvement	Yes	21	15	22 [16–28]	22	13	p=0.28
	No	17	8	30 [22–38]	21	56	Reference
CNS involvement	Yes	6	5	11 [6–16]	11	3	p=0.047
	No	32	18	30 [24–36]	30	30	Reference
Cervical LAP	Yes	30	20	23 [18–28]	21	56	p=0.041
	No	8	3	33 [25–41]	33	33	Reference
LDH level (continuous, Cox regression)	Median 600 (range 156–1790)	38	23	OS decreases with higher LDH	T-LBL median 660	B-LBL median 445	HR 1.12 (95% CI 1.04–1.21), p=0.004

Auto-SCT, autologous hematopoietic stem cell transplantation; Allo-SCT, allogeneic hematopoietic stem cell transplantation; LAP, lymphadenopathy; CNS, central nervous system; OS, overall survival; LDH, lactate dehydrogenase. P-values calculated using the log-rank test.

**Figure 2:**
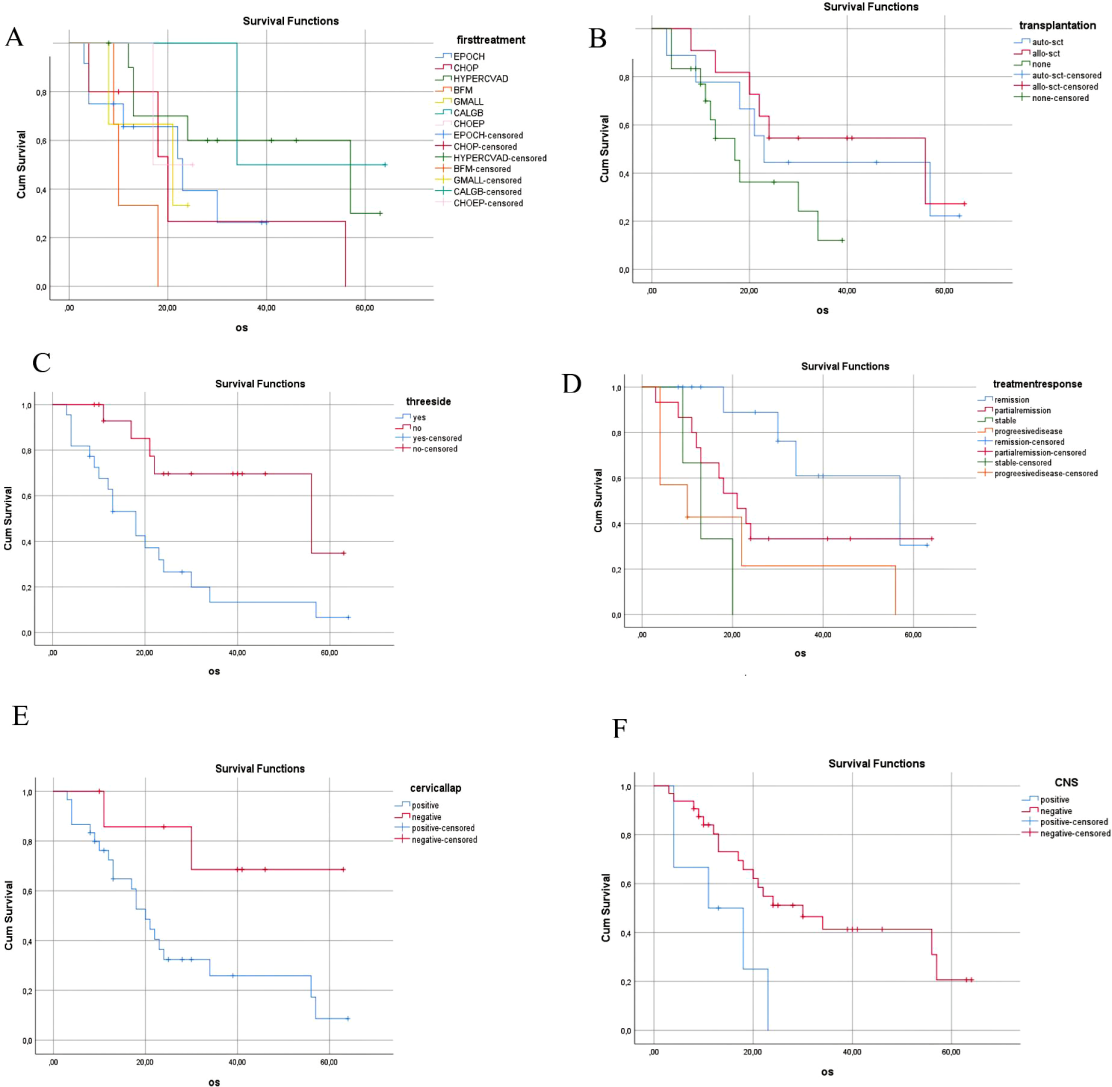
Kaplan–Meier overall survival (OS) curves according to clinical parameters in adult LBL patients. **(A)** OS by first-line treatment regimen. **(B)** OS by transplantation status. **(C)** OS by involvement of ≥3 anatomical regions. **(D)** OS by treatment response category. **(E)** OS by cervical lymphadenopathy status. **(F)** OS by CNS involvement. Censored cases are indicated by tick marks. Legends and axis labels have been enlarged for clarity.

Subgroup analyses of overall survival according to clinical parameters are presented in **Figure 2**, including treatment regimen, transplantation status, involvement of ≥3 anatomical regions, treatment response, cervical LAP, and CNS involvement.

Analysis of LDH levels among the 38 patients revealed a median value of 600 (range, 156–1790). The median LDH level was 660 in patients with T-LBL and 445 in those with B-LBL. Although no significant difference in LDH values was observed between the two groups, elevated LDH levels were significantly associated with inferior OS (p<0.05).

## Discussion

To our knowledge, this study represents the first multicenter national series of adult LBL patients from Türkiye, conducted between 2018 and 2024, providing country-level data on clinical characteristics, treatment patterns, and survival outcomes. The 5-year overall survival (OS) rate was 6% (**Figure 1A**). Our findings demonstrate that treatment approaches varied according to patient condition and immunophenotypic markers. Exploratory analyses suggested that elevated LDH levels, CNS involvement, disease affecting ≥3 anatomical regions, cervical lymphadenopathy, failure to achieve remission after initial therapy, and male gender (with no significant difference observed in the T-LBL subgroup) may correlate with inferior prognosis. Given the small sample sizes and univariate nature of these analyses, these results should be considered hypothesis-generating rather than definitive. Treatment approaches varied according to patient condition and immunophenotypic markers. Given the heterogeneity of regimens and center-specific practices, these data should be interpreted as descriptive rather than comparative.

The median OS in our cohort was 23 months and median progression-free survival (PFS) was 6 months. In comparison, Chen et al. reported a median OS of 37.0 months and PFS of 27.1 months in 43 patients with T-LBL in China (16). Consistent with our findings, their study confirmed the negative prognostic impact of elevated LDH, while also investigating circulating tumor DNA and genetic markers, which were not assessed in our cohort. CNS involvement was observed in 16% of patients in both studies and was consistently associated with poor outcomes. Unlike our study, Chen et al. did not evaluate the impact of lymph node involvement or bone marrow infiltration on survival. In our cohort, the 5-year OS rate was only 6%, reflecting the steep decline in survival after two years. This discrepancy between median OS and long-term survival highlights late events and limited durability of salvage therapies, and should be interpreted with caution.

In our study, mediastinal lymph node involvement was present in 89.5% of patients, pleural or pericardial effusion in 76%, and stage IV disease in 76%. These rates were comparable to those reported by Hoelzer et al. in 45 patients with T-LBL, where mediastinal involvement was 91%, effusion 40%, and stage III/IV disease 73% (17). Both studies demonstrated the adverse prognostic role of elevated LDH, while neither could establish superiority among treatment protocols. The median LDH value in our cohort was 604, slightly higher than in Hoelzer’s study, likely reflecting the higher proportion of advanced-stage patients. Unlike our findings, survival in advanced-stage patients was lower in Hoelzer’s cohort, possibly due to differences in patient selection.

Cortelazzo et al. investigated the role of minimal residual disease (MRD) in 30 patients with LBL (14). MRD positivity before transplantation reduced 5-year OS from 80% to 60%. Mediastinal involvement was observed in 84% of patients, similar to our cohort, while bone marrow involvement was 40% compared to 55% in our study. In both studies, bone marrow infiltration did not significantly affect survival. Unlike our cohort, only half of the patients were stage IV, and no CNS involvement was reported, which may explain their higher 5-year OS rate of 72%.

Although the retrospective design and limited sample size of our study restrict the strength of conclusions, the identification of potential prognostic factors such as CNS involvement, disease affecting ≥3 anatomical regions, cervical lymphadenopathy, and elevated LDH levels provides preliminary insight into risk stratification. These subgroup findings are illustrated in **Figure 2** illustrates exploratory subgroup findings suggesting that CNS involvement, cervical lymphadenopathy, and disease affecting ≥3 anatomical regions may be associated with inferior survival. Given the small sample size and reliance on univariate analyses, these associations should not be considered independent prognostic factors, but rather exploratory signals that may be confounded by overlapping clinical features. The observed association of cervical lymphadenopathy with inferior OS is unusual and may reflect confounding with advanced stage or elevated LDH rather than a true biological effect. These findings should be interpreted with caution and warrant validation in larger, prospective multicenter trials.

## Conclusion

This retrospective multicenter study provides descriptive data on adult patients with lymphoblastic lymphoma in Türkiye. To our knowledge, this represents one of the first national series, though prior unpublished work cannot be fully excluded. The heterogeneity of treatment protocols and limited sample size restrict definitive comparisons, yet the findings highlight clinical features and survival outcomes that merit further prospective validation. Future studies with larger cohorts and standardized treatment approaches are needed to strengthen evidence and guide clinical practice.

## Data Availability

The datasets presented in this study can be found in online repositories. The names of the repository/repositories and accession number(s) can be found below: All patient data were obtained from the hospital data archive developed by the Ministry of Health of the Republic of Türkiye. Due to institutional regulations, the datasets are not publicly available but can be made available to editors and reviewers upon reasonable request by contacting the corresponding author.
