# Supplementary material for: Clinicopathological features of adult lymphoblastic lymphoma: a retrospective multicenter study from Türkiye
Source: Front Oncol. 2026 May 18;16:1820823. doi: 10.3389/fonc.2026.1820823 (PMC13222830; doi:10.3389/fonc.2026.1820823)
Supplement: Supplementary file 1 [file Table1.docx]

**Supplementary materials**

**Supplementary Table S1.** Extent of missing data and exclusions from subgroup analyses. Ten patients had incomplete records: 5 with missing LDH values, 3 with incomplete staging information, and 2 with missing treatment response documentation. These patients were excluded from the respective subgroup analyses but retained in overall survival (OS) and progression‑free survival (PFS) calculations.

| **Variable / Analysis** | **Patients with missing data (n)** | **Reason for exclusion from subgroup analysis** | **Retained in OS/PFS calculations** |
| --- | --- | --- | --- |
| LDH level | 5 | Incomplete laboratory records | Yes |
| Staging (anatomical regions) | 3 | Missing imaging/staging information | Yes |
| Treatment response assessment | 2 | Missing documentation of remission/relapse | Yes |
| **Total excluded from subgroup analyses** | 10 |  | **All retained** |
